# Epidemiology of herpes zoster in National Guard Hospitals in Saudi Arabia: a 6-year retrospective chart review study

**DOI:** 10.3389/fpubh.2024.1479640

**Published:** 2025-02-20

**Authors:** Fayssal Farahat, Mohammed AlZunitan, Asim Alsaedi, Wafa Al Nassir, Ayman Elgammal, Syed Nazeer, Majid Althaqafy, Aiman El-Saed, Nouf Al Enizi, Sulafah Hakami, Zainab Alsharef, Adriana Guzman-Holst, Majid Alshamrani

**Affiliations:** ^1^Infection Prevention and Control Program, King Abdulaziz Medical City, King Saud bin Abdulaziz University for Health Sciences, King Abdullah International Medical Research Center, Ministry of National Guard Health Affairs, Riyadh, Saudi Arabia; ^2^Infection Prevention and Control Department, King Abdulaziz Medical City, King Saud bin Abdulaziz University for Health Sciences, King Abdullah International Medical Research Center, Ministry of National Guard Health Affairs, Jeddah, Saudi Arabia; ^3^Infection Prevention and Control Department, Imam Abdulrahman Al Faisal Hospital, Ministry of National Guard Health Affairs, Dammam, Saudi Arabia; ^4^Infection Prevention and Control Department, King Abdulaziz Hospital, Ministry of National Guard Health Affairs, Alahsaa, Saudi Arabia; ^5^Infection Prevention and Control Department, Prince Mohammed Bin Abdulaziz Hospital, Ministry of National Guard Health Affairs, Al Madinah, Saudi Arabia; ^6^GSK Vaccines, Jeddah, Saudi Arabia; ^7^GSK Vaccines, Wavre, Belgium

**Keywords:** herpes zoster, Saudi Arabia, epidemiology, post-herpetic neuralgia, comorbidity

## Abstract

**Introduction:**

Incidence of herpes zoster (HZ) is increasing worldwide, imposing significant burden on healthcare resources. In Saudi Arabia, local epidemiological studies are limited, and HZ burden is unknown.

**Methods:**

This multi-center, hospital-based, retrospective medical chart review was conducted at five National Guard hospitals and affiliated primary care centers. Patients included military personnel, healthcare workers, and family dependents, in addition to non-eligible individuals via referral from other healthcare systems. Data were retrospectively collected from electronic medical records of documented cases of HZ or related complications from January 2017–December 2022.

**Results:**

1,019 HZ cases were identified, with the number of cases increasing annually (2017: 89; 2022: 279). Estimated HZ prevalence over the study period was 0.12%. Mean age of patients was 52.8 years and >50% were females. Most (73.9%) patients had ≥1 comorbidity, most commonly hypertension (38.9%) and diabetes (37.7%). HZ-related complications were detected in 31.3% of cases; post-herpetic neuralgia was diagnosed in 17.6% and disseminated HZ in 5.6% of patients. In total, 12.5% of patients were hospitalized; 1.2% required intensive care unit admission. Mean hospital stay was 10.1 days. Use of antiviral medications was reported in most cases (87.5%). Significant predictors of complicated HZ, identified via multivariable logistic regression analyses, were age ≥60 years (odds ratio=1.42; *p*=0.03), autoimmune disease (2.45; *p*<0.01), depression (2.68; *p*=0.02), and chronic lung disease (1.95; *p*=0.04).

**Conclusion:**

This study provides updated insights into HZ epidemiology in Saudi Arabia. A high proportion of patients identified in a hospital setting with HZ had comorbidities and a substantial proportion experienced complications.

## 1 Introduction

Herpes zoster (HZ), commonly known as shingles, is a viral disease caused by the reactivation of the varicella-zoster virus which primarily presents as a painful vesicular skin rash (1, 2). Although HZ is usually self-limiting, it may also result in complications such as chronic pain in the area of the initial rash (post-herpetic neuralgia, PHN) which occurs in 5–30% of patients with HZ (2, 3). Other complications of HZ include ocular involvement with visual impairment and disseminated HZ in immunocompromised individuals (2, 3). Both HZ and its complications are associated with reduced quality of life and increased healthcare costs (4, 5).

The global incidence of HZ is increasing, with a systematic literature review of HZ incidence in the general population aged ≥50 years reporting an incidence rate ranging from 5.2 to 10.9 cases per 1,000 person-years (6). Older adult populations are particularly at risk of HZ infection due to a reduction in cellular immunity, with annual reported incidences of HZ increasing with age after ≥50 years and peaking in those aged 70–79 years (7, 8). Therefore, as the global population ages, an increased HZ burden is likely. Additionally, reduced immune function and other underlying conditions such as diabetes, chronic obstructive pulmonary disease (COPD), chronic kidney disease, and rheumatic disease can also increase the risk of HZ infection (9–11). For example, a recent meta-analysis reported a relative risk of HZ of 1.4 in patients with COPD, with additional data indicating that inhaled corticosteroids, a common treatment for COPD, may be a separate risk factor for HZ in this population (10, 12). Additionally, a 2023 observational study of a medical insurance database in Taiwan found a greater incidence of HZ in patients with rheumatoid arthritis than those without (incidence rate ratio: 1.7), with treatments for rheumatoid arthritis also being associated with higher risk of HZ (13).

HZ vaccines, such as the recombinant zoster vaccine, have been shown to effectively prevent HZ and PHN, and can alleviate the overall burden and disease complications associated with HZ and PHN (5, 6, 14). For example, implementing HZ vaccination into national immunization programs has been associated with long-term, substantial reductions in incidence of HZ in multiple countries, including England and Australia (15, 16). Despite the availability of effective vaccines against HZ, global uptake is limited due to several factors, including lack of awareness of the burden of HZ, and vaccines not being included in national immunization programs in many developing countries (17).

In Saudi Arabia, despite HZ vaccination being available free of charge since 2022 for individuals aged ≥50 years and individuals aged ≥18 years with immunocompromising conditions (18), uptake is particularly low. This is in part due to low public awareness of HZ complications and its vaccination in Saudi Arabia, with a recent study reporting that only 29.6% of participants recognized the risk of HZ following chickenpox infection (19). Another reported that only 7.8% of participants had a high level of knowledge regarding HZ, with even fewer participants having adequate knowledge of HZ vaccination (3.1%) (20). For individuals aware of HZ vaccination, uptake remains low, with multiple cross-sectional studies of adults aged ≥50 years conducted in 2023 reporting that although 55.8–57.2% of participants had heard of an HZ vaccine, only 5.4–7.7% had received it (21, 22).

Furthermore, in many countries, including Saudi Arabia, epidemiological studies are scarce due to limited routine surveillance of this disease, with HZ only recently being included in the list of mandatory notifiable diseases (23). As a result, HZ is often under-reported and therefore the burden of HZ in Saudi Arabia remains unknown. A review of the literature reporting on the epidemiology of HZ in the Gulf Cooperation Council countries (which includes Saudi Arabia) identified a clear lack of evidence for the incidence of HZ infection, demonstrating that further research is needed to inform the development of HZ vaccination programs in these countries (24).

**Figure 1:**
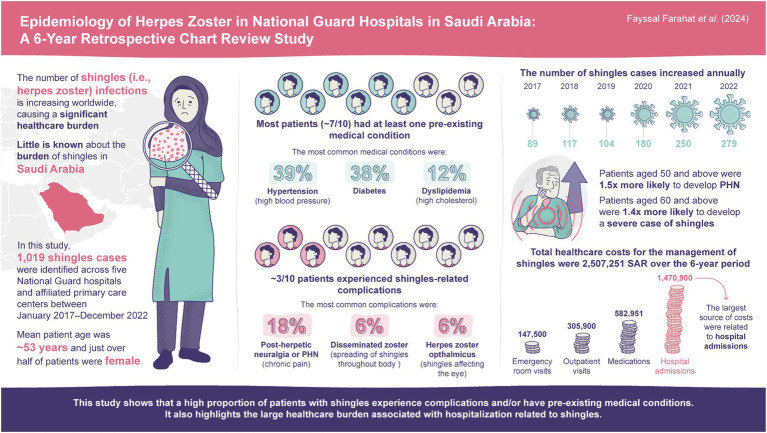
Graphical abstract. PHN, post-herpetic neuralgia; SAR, Saudi Riyal.

In this retrospective electronic medical chart review, we estimate the prevalence of HZ and examine the epidemiological and clinical characteristics of HZ cases in outpatient and inpatient settings in the Ministry of National Guard Health Affairs (MNGHA) hospitals and affiliated Primary Healthcare Centers (PHCs) in Saudi Arabia. We also describe HZ clinical management and the patient pathway, and estimate direct costs and resource utilization associated with HZ treatment, hospitalization, and medical visits. A graphical abstract summarizing the key conclusions from this manuscript is available in Figure 1.

## 2 Methods

This multi-center hospital-based retrospective electronic medical chart review was conducted at the MNGHA hospitals in Saudi Arabia, which form part of the government healthcare system managed by the Corporate Health Affairs. Data from five MNGHA hospitals from the Central, Western, and Eastern regions were reviewed: Riyadh (1,796 beds), Jeddah (746 beds), Alahsaa (305 beds), Almadinah (331 beds), and Dammam (161 beds). A total of 71 Family Medicine Centers and PHCs are affiliated with these five hospitals. These hospitals and PHCs provide medical services to the entire local population including children, adolescents, adults, and older adults (25). Although the hospitals provide secondary and tertiary care, family medicine services are provided primarily via the PHCs.

### 2.1 Study population

The study population included National Guard eligible individuals of all ages who are military personnel, and healthcare workers and their family dependents, in addition to non-eligible individuals granted eligibility based on referral from other healthcare systems. To estimate HZ burden in this population, we assessed the number of clinically diagnosed cases of HZ and related complications across the five MNGHA hospitals and 71 PHCs, for both hospitalized inpatients and outpatients requiring ambulatory consultations over a 6-year study period (January 2017–December 2022).

### 2.2 Primary outcomes

The primary outcome aimed to estimate the frequency and prevalence of HZ and associated complications. Data were collected on demographic information including age (in years), comorbidities, HZ clinical diagnosis, HZ-related complications, hospitalization, outpatient visits, and medications. These data were gathered using the BESTCare electronic medical record system, which is implemented in all MNGHA hospitals and PHCs.

Cases of HZ and HZ-related complications were identified by searching for the following International Classification of Diseases 10^th^ Revision (ICD-10) diagnosis codes within the BESTCare records: HZ without complications (B02.9), encephalitis due to HZ (B02.0), meningitis due to HZ (B02.1), HZ with nervous system complications (B02.2), ocular HZ (B02.3), disseminated HZ (B02.7), HZ with other complications (B02.8), and PHN (G53.0). HZ cases are also reported internally within MNGHA healthcare facilities to the Infection Prevention and Control (IPC) Department in each region. Therefore, to validate data quality and accuracy of coding and reporting, both BESTCare and separate reports from the IPC Department were verified for number of reported cases. Although we assume that some mild cases may not be reported to the IPC Department, each diagnosis is documented on BESTCare and is therefore identifiable through the medical records department.

PHN was defined as pain persisting for 90 days after onset of the HZ-related rash. The diagnosis of HZ-related encephalitis and meningitis was based on clinical history, examination, imaging (magnetic resonance imaging and/or computed tomography scan), cerebrospinal fluid analysis, and identification of the pathogen in cerebrospinal fluid by polymerase chain reaction (PCR) amplification. Ocular HZ was diagnosed based on clinical history and examination.

### 2.3 Secondary outcomes

The secondary outcome aimed to measure healthcare resource utilization associated with management of HZ and HZ-related complications and estimate the direct medical costs (in Saudi Riyal, SAR). Costs included medications, hospitalizations, outpatient visits, and related follow-up appointments. Details of each patient’s treatment pathway (including admissions, diagnosis, and medications) were extracted from their respective BESTCare electronic medical record.

Initial diagnosis of HZ cases occurs mostly at the emergency department or ambulatory care clinics. Disseminated HZ or severe complications usually require hospital admission, while mild to moderate cases receive outpatient management with a follow-up with family medicine, dermatology, neurology, or other specialty depending on the severity and affected area. Clinical management at the MNGHA is free-of-charge for all eligible patients, and non-eligible patients can still receive a free-of-charge service following Medical Eligibility Committee approval. Other non-eligible patients are covered through their own medical insurance, and some may pay out-of-pocket. Therefore, the cost analysis was performed from the perspective of the payer (the MNGHA) and data on unit costs (i.e., the cost of one unit of healthcare service such as an outpatient or emergency department visit) were sourced from the MNGHA Business Center, which is based on the national tender from the National Unified Procurement Company (NUPCO). NUPCO is responsible for centralizing the procurement, logistics, and supply chain management for pharmaceutical, medical devices, and supplies for governmental hospitals in Saudi Arabia.

### 2.4 Statistical analysis

Data were analyzed using IBM SPSS v28. Descriptive statistics included frequency of HZ cases, HZ-related complications, and hospitalizations (stratified by age, sex, and co-morbidities). The normality assumption of data was assessed using the Shapiro-Wilk test. Data are considered normally distributed with *p* value >0.05. For data that were not normally distributed, the median and interquartile range (IQR) were calculated alongside the means to provide a comprehensive overview of data distribution.

The prevalence of HZ over the study period was estimated using the average annual number of active medical records in the studied hospitals during the whole study period (N=827,746) as the average catchment population for study period:


$$\frac{\begin{array}{c} \mathrm{Total}\;\mathrm{number}\;\mathrm{of}\;\mathrm{existing}\;HZ\;\mathrm{cases}\;\mathrm{identified}\\ \;\mathrm{during}\;a\;\mathrm{specified}\;\mathrm{time}\;\mathrm{interval} \end{array}}{\begin{array}{l} \mathrm{Average}\;\mathrm{catchment}\;\mathrm{population}\;\\ \mathrm{during}\;\mathrm{the}\;\mathrm{same}\;\mathrm{time}\;\mathrm{interval} \end{array}}x\;100$$

If applicable, a proxy of the cumulative incidence was estimated as:


$$\frac{\begin{array}{c} \mathrm{Total}\;\mathrm{number}\;\mathrm{of}\;\mathrm{cases}\;\mathrm{during}\;\\ \mathrm{specified}\;\mathrm{time}\;\mathrm{interval} \end{array}}{\begin{array}{l} \mathrm{Average}\;\mathrm{catchment}\;\mathrm{population}\;\\ \mathrm{during}\;\mathrm{the}\;\mathrm{same}\;\mathrm{time}\;\mathrm{interval} \end{array}}\mathrm{per}\;1,000\;\mathrm{patients}$$

Subsequent episodes of HZ in the same patient were not included in these calculations.

A univariate analysis was carried out to identify variables for multivariable logistic regression models, including potential risk factors, along with other variables of known clinical relevance. These multivariable logistic regression (LR, backward stepwise) analyses used odds ratios (ORs) and 95% confidence intervals (CIs) and were performed to determine significant risk factors associated with HZ-related complications. Level of significance was determined at *p*<0.05.

Costs of hospitalization were calculated per night (single vs. shared room) in regular ward, airborne or contact isolation room, or ICU admission. Physician visit during hospitalization was calculated based on a single visit daily by the main responsible physician (MRP). Costs of outpatient visits were also calculated and varied according to physician specialty. Both costs of outpatient and emergency department visits were estimated per patient. Medication costs were calculated based on the Business Center list of formulary medications that matched public prices.

Overall cost was calculated by totaling the cost of hospitalization per night, inpatient physician visit, outpatient physician visit, and total cost of related medications per patient. The median (IQR) and mean (standard deviation, SD) of each cost category was calculated for the study population.

### 2.5 Ethical compliance

The study was conducted in accordance with applicable subject privacy requirements and the guiding principles of the Declaration of Helsinki. Approval of the Institutional Review Board at King Abdullah International Medical Research Center was obtained to conduct the study (SRC22R/007/04).

## 3 Results

### 3.1 Demographics and clinical characteristics

A total of 1,019 HZ cases were identified during the 6-year study period (January 2017–December 2022; Table 1). Over half of the HZ cases were in females (51.2%), and mean (SD) and median (IQR) age was 52.8 (18.3) and 55.0 (38.0–66.0) years, respectively; 6.1% of cases were in adults ≥80 years. A total of 74.6% of cases were dependents, 15.4% were military personnel, and 10.0% were healthcare workers.

**Table 1:** Demographic and clinical characteristics of the study population.

Variables	*N*=1,019
Age; years	
Mean	52.8
Median (IQR)	55.0 (38.0–66.0)
Sex; *n* (%)	
Female	522 (51.2)
Male	497 (48.8)
Occupation; *n* (%)	
Healthcare worker	102 (10.0)
Military	157 (15.4)
Dependents	760 (74.6)
Comorbidities; ^a^ *n* (%)	
Hypertension	396 (38.9)
Diabetes mellitus	384 (37.7)
Dyslipidemia	126 (12.4)
Chronic heart disease	109 (10.7)
Cardiovascular disease	90 (8.8)
Coronary artery disease	33 (3.2)
Chronic kidney disease	82 (8.0)
Lung disease^b^	98 (9.6)
Asthma	74 (7.3)
COPD	9 (0.9)
Chronic lung disease	38 (3.7)
Malignancy	71 (7.0)
Hematopoietic stem cell	18 (1.8)
Organ transplant	23 (2.3)
Rheumatoid arthritis	35 (3.4)
Inflammatory bowel disease	16 (1.6)
Other autoimmune disease	44 (4.3)
Immunosuppressive medications^c^	122 (12.0)
Depression	24 (2.4)
Recent COVID-19 infection^d^	87 (8.5)
Number of comorbidities per patient; *n* (%)	
1	270 (26.5)
2	211 (20.7)
≥3	272 (26.7)
History of previous HZ disease;^e^ *n* (%)	31 (3.0)
History of varicella infection; *n* (%)	
Yes	345 (33.9)
Unknown	674 (66.1)
History of varicella vaccination; *n* (%)	
Yes	72 (7.1)
No/Unkown	947 (92.9)

No cases of HIV were reported. ^a^HIV is not routinely tested for in Saudi Arabia; ^b^Included asthma, COPD, and other chronic lung conditions; ^c^Most common immunosuppressive medications: chemotherapy (*n*=33), methotrexate (*n*=9), azathioprine (*n*=8), cyclosporine (*n*=5), prednisolone (*n*=6), tacrolimus (*n*=6); ^d^Median time of HZ infection following COVID-19 infection=261 days (IQR=124–468 days); ^e^Recurrent cases counted individually. COPD, chronic obstructive pulmonary disease; COVID-19, coronavirus disease 2019; HIV, human immunodeficiency virus; HZ, herpes zoster; IQR, interquartile range; SD, standard deviation.

**Figure 2:**
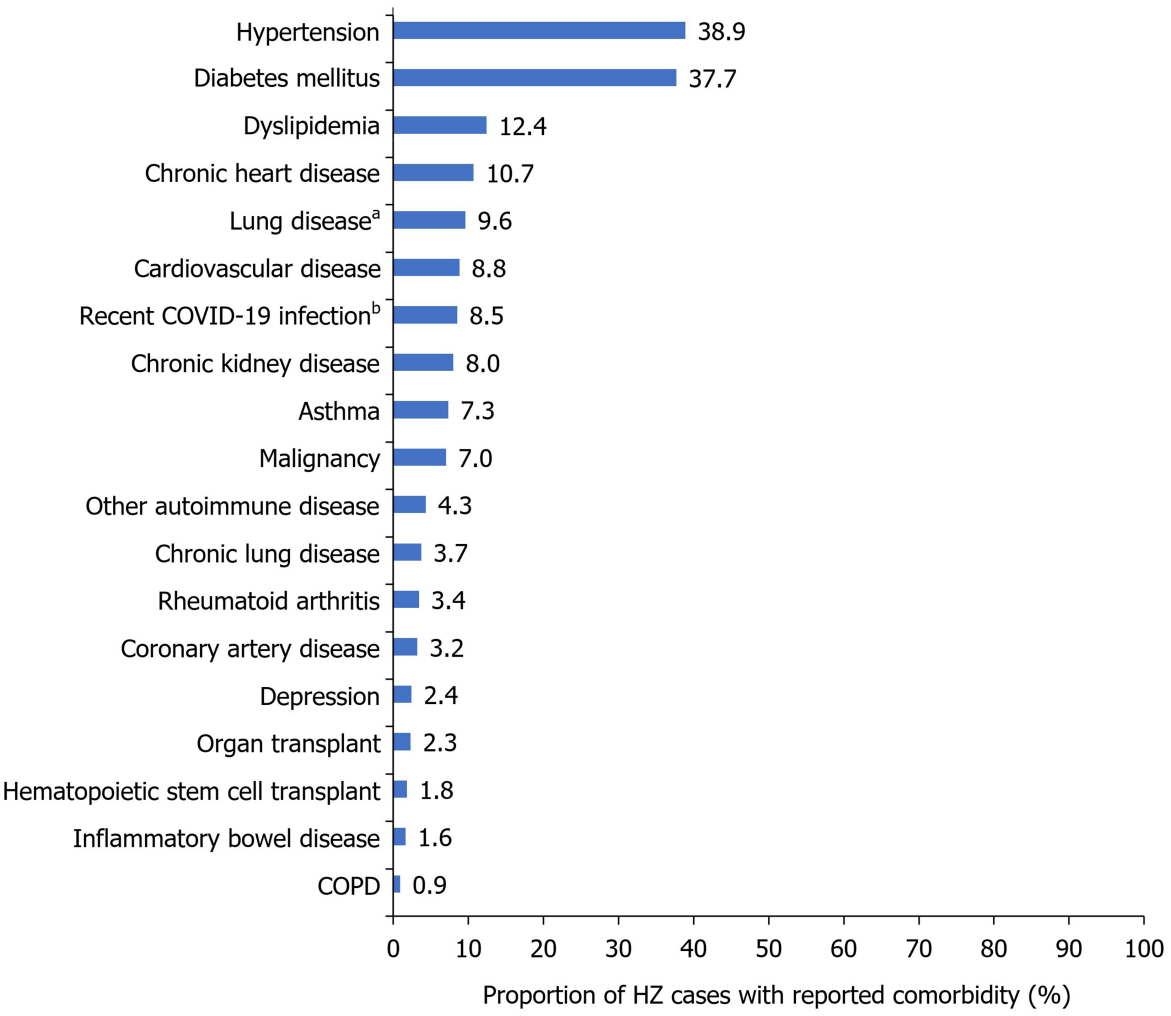
Frequency of HZ comorbidities within the study population. Reported as a proportion of the total number of HZ cases during the study period (2017–2022; *N*=1,019). ^a^Included asthma, COPD, and other chronic lung conditions; ^b^Median time to HZ infection following COVID-19=216 days (IQR=124–468 days). COPD, chronic obstructive pulmonary disease; COVID-19, coronavirus disease 2019; HZ, herpes zoster; IQR, interquartile range.

The majority of patients had ≥1 comorbidity (73.9%) and almost one-third had ≥3 comorbidities (26.7%; Table 1). The most common comorbidities were hypertension (38.9%), diabetes mellitus (37.7%), dyslipidemia (12.4%), and chronic heart disease (10.7%; Figure 2). A total of 21.2% (*n*=216) of patients had a medical history indicative of a compromised immune system (chronic kidney disease, malignancy, hematopoietic stem cell transplant, organ transplant, rheumatoid arthritis, and other autoimmune diseases); including some patients with ≥1 immunocompromising condition. A history of previous HZ infection was identified in only 3.0% of cases (Table 1). However, approximately two-thirds (66.1%) of patients were unaware of their history of previous varicella infection. Moreover, history of vaccination against varicella was reported by a small proportion of the studied patients (7.1%).

### 3.2 Prevalence and incidence of HZ

**Figure 3:**
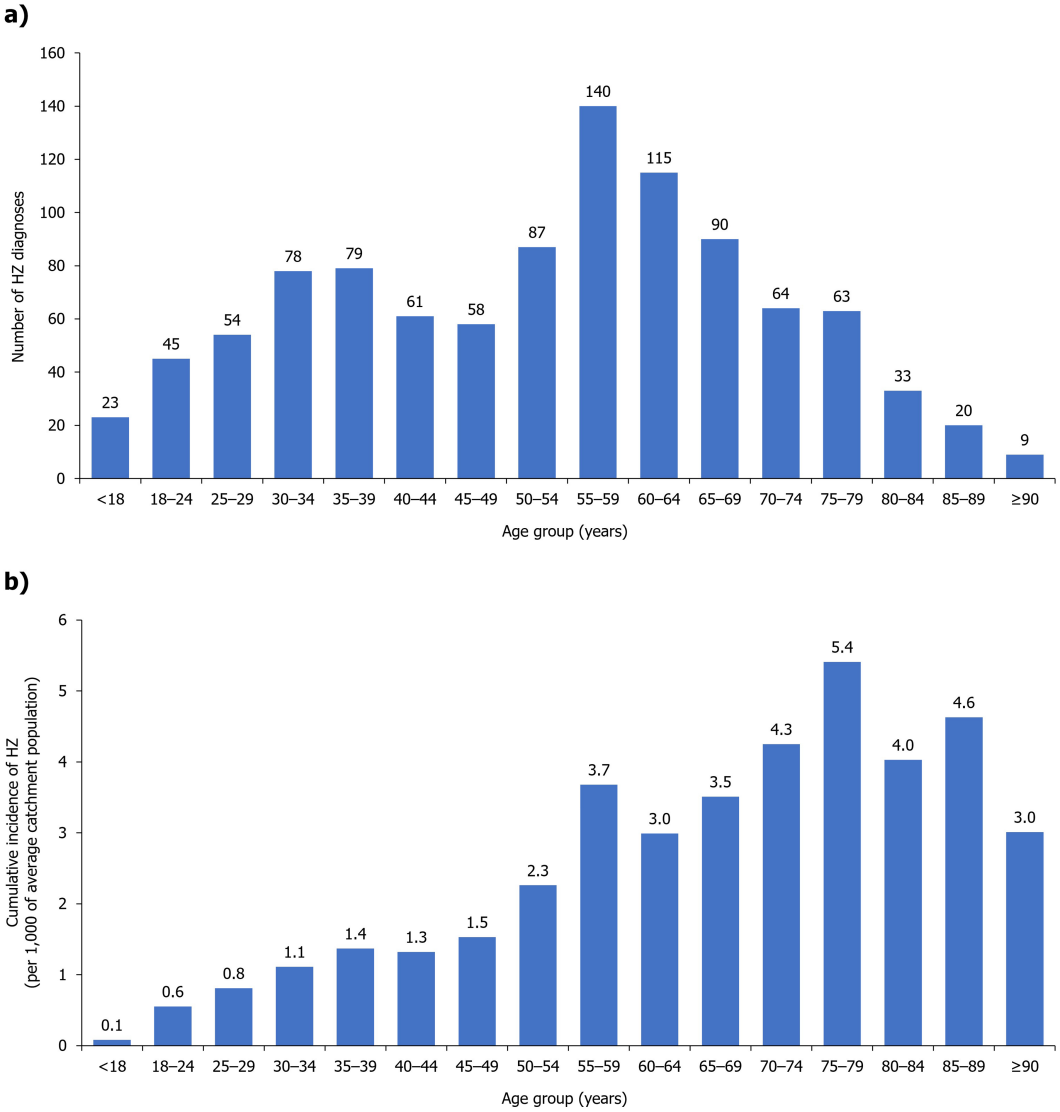
**(A)** Frequency and **(B)** cumulative incidence of HZ cases per age group during the study period (2017–2022). HZ, herpes zoster.

The estimated prevalence of HZ over the total study period was 0.1%, which translates to a cumulative incidence of 1.2 per 1,000 MNGHA population. HZ cumulative incidence gradually increased with age and was the highest in patients aged 75–79 years old (5.4 per 1,000 MNGHA population), with a range of 2.3–5.4 per 1,000 MNGHA population among individuals aged ≥50 years (Figure 3). Prevalence and cumulative incidence of HZ per age group over the study period is summarized in Supplementary Table 1.

**Figure 4:**
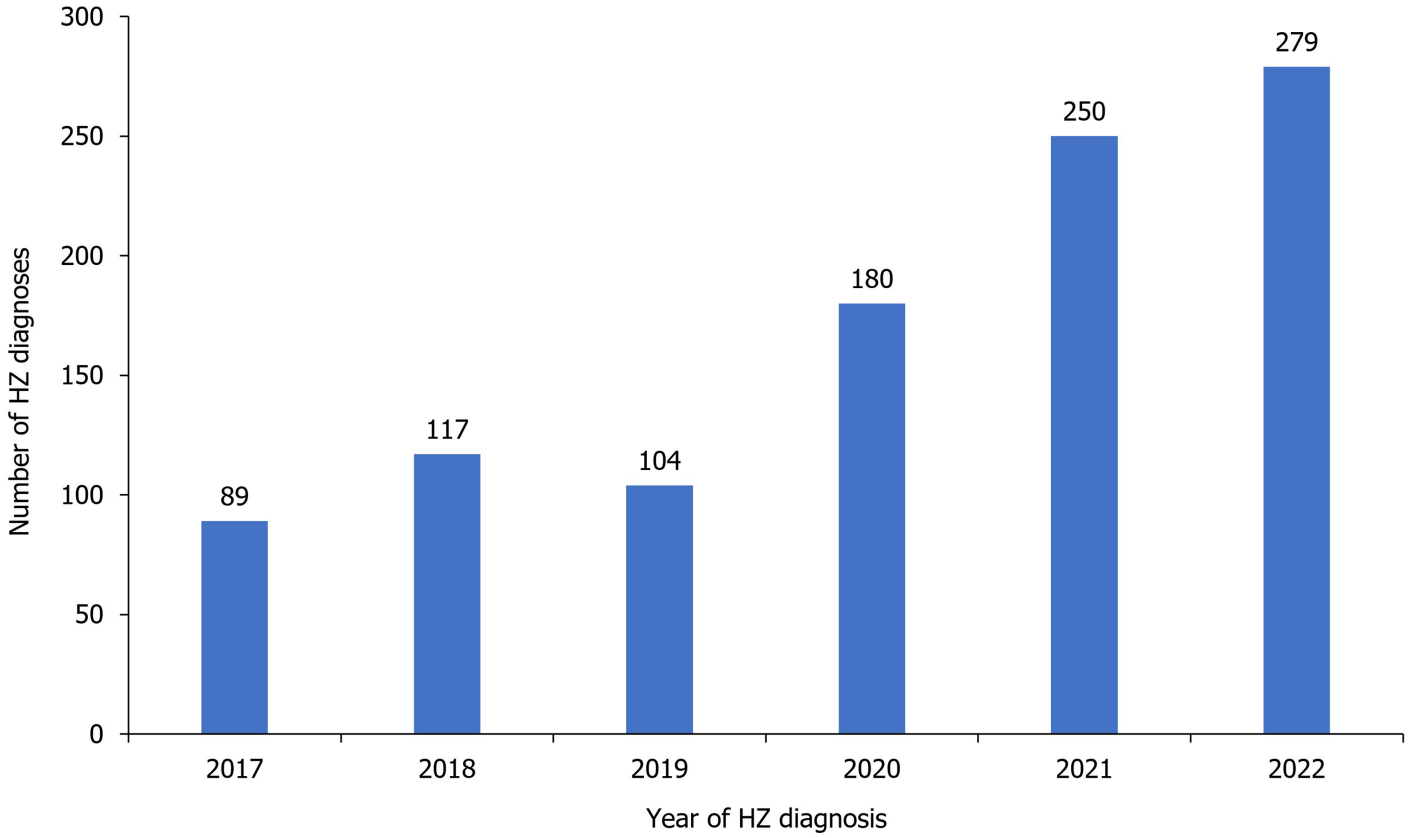
Distribution of HZ diagnoses per year during the study period. HZ, herpes zoster.

Number of annual reported HZ cases remained similar from 2017 (n=89) to 2019 (n=104), before increasing to reach 279 cases in 2022 (Figure 4, Table 2). Most cases were diagnosed in hospital emergency departments (59.6%) followed by outpatient departments (34.4%). Most cases were diagnosed clinically (97.4%; Table 2).

**Table 2:** HZ disease diagnosis and clinical characteristics.

Variables	*N*=1,019
Case diagnosis per study year; *n* (%)	
2017	89 (8.7)
2018	117 (11.5)
2019	104 (10.2)
2020	180 (17.7)
2021	250 (24.5)
2022	279 (27.4)
Type of diagnosis; *n* (%)	
Clinical	992 (97.4)
Laboratory^a^	27 (2.6)
Place of HZ diagnosis; *n* (%)	
Emergency department	607 (59.6)
Hospital outpatient	351 (34.4)
Primary Healthcare Center	31 (3.0)
Hospital inpatient	30 (2.9)
Most common diagnosing physician; *n* (%)	
Emergency department	248 (24.3)
Family Medicine	365 (35.8)
Dermatology	173 (17.0)
Internal Medicine/Infectious Diseases	103 (10.1)
Neurology	47 (4.6)
Type of rash at time of diagnosis; *n* (%)	
Erythematous	891 (87.4)
Pustular	246 (24.1)
Crusted	135 (13.2)
Hemorrhagic	30 (2.9)
Dermatome; *n* (%)	
Single dermatome	888 (87.1)
Multi-dermatome	131 (12.9)
Dermatome distribution; *n* (%)	
Thoracic	475 (61.2)
Cranial	99 (12.8)
Lumbar	73 (9.4)
Cervical	116 (14.9)
Sacral	13 (1.7)
Symptoms; *n* (%)	
Burning pain	854 (83.8)
Throbbing pain	95 (9.3)
Stabbing pain	160 (15.7)
Headache	106 (10.4)
Fever	106 (10.4)
Fatigue	117 (11.5)
Patients with complicated HZ; *n* (%)	319 (31.3)
Types of complicated HZ; *n* (%)	
PHN	179 (17.6)
Disseminated HZ	57 (5.6)
Ocular HZ^b^	57 (5.6)
HZ oticus	14 (1.4)
Encephalitis	8 (0.8)
Meningitis	8 (0.8)
Other nervous system complications	14 (1.4)
Secondary bacterial infection	17 (1.7)

^aLaboratory diagnosis included polymerase chain reaction, culture, and direct fluorescent antibody; bFive cases had acute retinal necrosis and 25 cases suffered from blurring of vision. HZ, herpes zoster; PHN, post-herpetic neuralgia.^

### 3.3 HZ presentation and related complications

At time of diagnosis, most patients with HZ presented with an erythematous rash (87.4%) followed by a pustular rash (24.1%; Table 2). Most had a single affected dermatome (87.1%), with thoracic being the most common distribution (61.2%). Burning pain was reported in 83.8% of cases.

**Figure 5:**
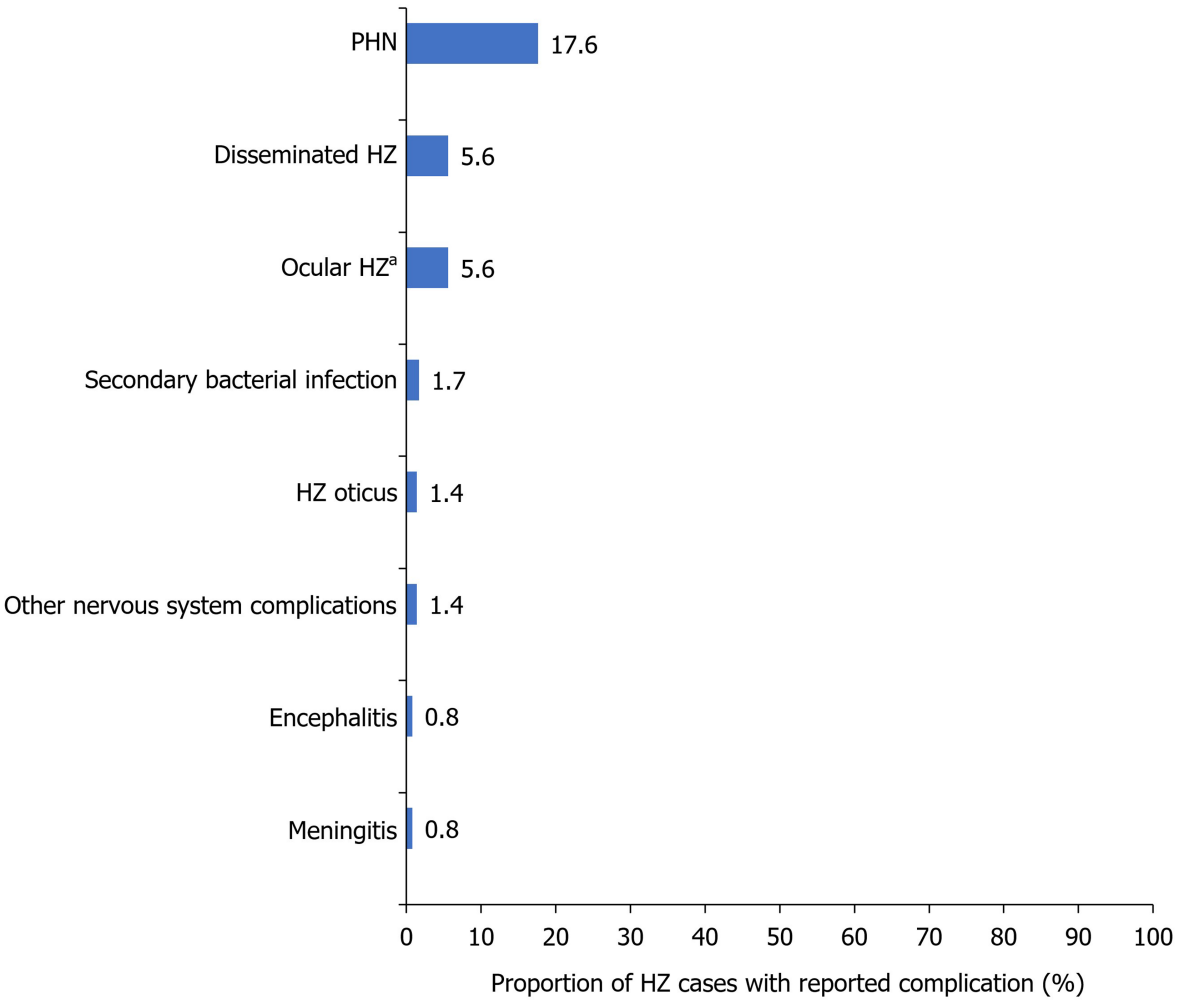
Frequency of HZ-related complications within the study population. Reported as a proportion of the total number of HZ cases during the study period (2017–2022; *N*=1,019). ^a^Five cases had acute retinal necrosis and 25 cases suffered from blurring of vision. HZ, herpes zoster; PHN, post-herpetic neuralgia.

HZ-related complications were detected in 31.3% of cases, with PHN being diagnosed in 17.6%, disseminated HZ in 5.6%, and ocular HZ in 5.6% of cases (Table 2, Figure 5).

### 3.4 HZ disease management

Overall, 12.5% of HZ cases were admitted to a hospital, with 9.1% requiring airborne isolation (Supplementary Table 2). Mean and median length of hospital stay were 10.1 days and 6.0 days (IQR: 2.0–14.0), respectively.

Use of antiviral medications were reported in majority of cases (87.5%) with the most common being acyclovir (51.4%) followed by famciclovir (35.5%). Systemic antibiotics were prescribed in only 4.1% of cases (Supplementary Table 2).

### 3.5 HZ and associated risk analysis

**Figure 6:**
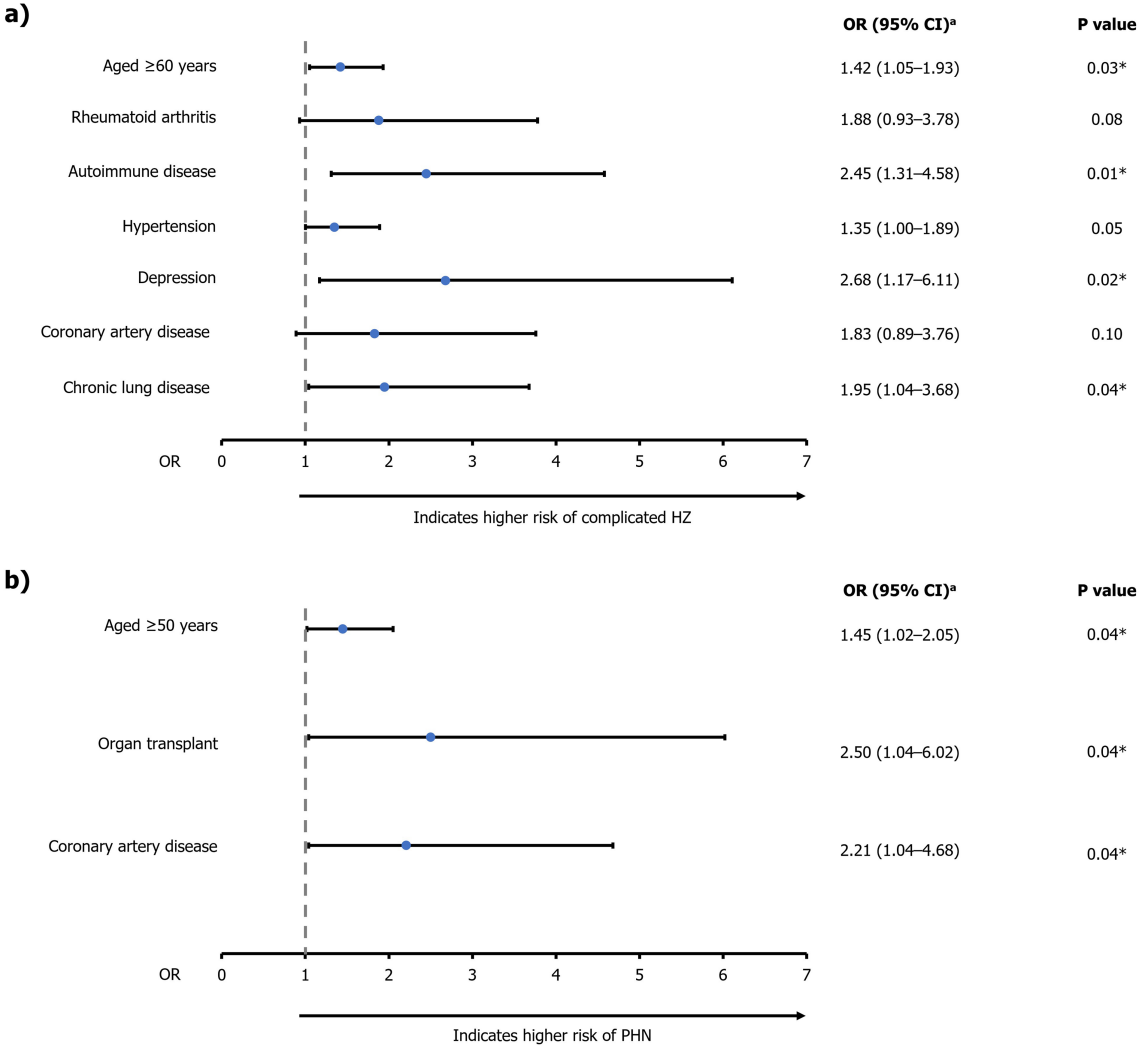
Multivariable logistic regression analysis of factors associated with **(A)** complicated HZ and **(B)** PHN. ^a^Estimated using backward logistic regression method. *Significant predictors (*p*<0.05) of **(A)** complicated HZ or **(B)** PHN. For risk of PHN, only significant predictors are presented due to the limited sample size of patients with PHN (*n*=179). CI, confidence interval; HZ, herpes zoster; OR, odds ratio; PHN, post-herpetic neuralgia.

Significant predictors of complicated HZ disease (OR [95% CI]) were age ≥60 years (1.42 [1.05–1.93]; *p*=0.03), autoimmune disease (2.45 [1.31–4.58]; *p*<0.01), depression (2.68 [1.17–6.11]; *p*=0.02), and chronic lung disease (1.95 [1.04–3.68]; *p*=0.04; Figure 6; Supplementary Table 3). Significant predictors of PHN (OR [95% CI]) were age ≥50 years (1.45 [1.02–2.05]; *p*=0.04), organ transplant (2.50 [1.04–6.02]; *p*=0.04), and coronary artery disease (2.21 [1.04–4.68]; *p*=0.04) (Figure 6; Supplementary Table 4).

### 3.6 Healthcare resource utilization and cost analysis

The total number of outpatient and emergency department visits related to HZ in the study period were 1,266 and 671, respectively (Table 3). There were a total of 127 hospital admissions and 990 patients had documented prescribed medications. These utilization values were used to estimate costs.

**Table 3:** HZ-related healthcare resource utilization by unit.

Healthcare resource	Utilization (*n*)	Total cost (SAR)
Outpatient visits^a^	1,266	305,900
Emergency department visits	671	147,500
Hospital admission^b^	127	1,470,900
Medical prescriptions	990	582,951
**Overall cost for study population (*N*=1,019)**		**2,507,251**

^a^Total number of outpatient visits, including those who were initially diagnosed in the outpatient department (includes PHC), follow-ups and all visits; ^b^Includes intensive care unit admissions and airborne isolation. HZ, herpes zoster; OPD, outpatient department; PHC, Primary Healthcare Center; SAR, Saudi Riyal.

From the perspective of the payer (the MNGHA), the total overall direct costs in the study period related to HZ (*n*=1,019) were 2,507,251 SAR (Supplementary Table 5). When considering all patients with HZ identified, mean direct cost per patient with HZ (per episode) was 2,461 SAR (approximately 656 United States dollars [USD], using an exchange rate of 1 USD=3.75 SAR (26)). Median (IQR) direct cost per patient with HZ was 927 SAR (507–1,636). The largest source of costs among patients were related to hospital admissions (total cost: 1,470,900 SAR; mean [median] cost per hospitalized patient with HZ: 11,582 [6,000] SAR).

Total direct costs related to HZ disease with PHN (*n*=179) were 361,047 SAR (Supplementary Table 5), with the largest source of costs also being hospital admissions (162,900 SAR). Among patients with complicated HZ (*n*=319), the total direct costs represented almost half of the overall patient costs (1,240,512 SAR). Furthermore, the mean cost per patient with complicated disease was more than twice the mean cost for the uncomplicated cases (3,889 vs. 1,810 SAR; Supplementary Table 5). Median costs per patient were 1,239 and 877 SAR, respectively.

## 4 Discussion

This multi-center, hospital-based, retrospective medical chart review of MNGHA hospitals provides updated insights into HZ epidemiology in Saudi Arabia. The study showed that the majority of patients with HZ were of an older age (mean: 52.8 years), consistent previous studies and likely due to declining HZ-specific cell-medicated immunity which progresses with age (27–31). The study also showed a high prevalence of several chronic diseases among the study population (e.g., hypertension, diabetes mellitus, dyslipidemia, chronic lung disease, chronic kidney disease, and cardiovascular illnesses), which is consistent with a previous retrospective study of HZ epidemiology in a primary care setting in Saudi Arabia (31).

The number of HZ cases generally increased annually over the study period, from 89 cases in 2017 to 279 cases in 2022. Potential explanations for this increase may be improved reporting and a greater awareness of HZ amongst physicians, and the coronavirus disease 2019 (COVID-19) pandemic, with there being several recent reports of HZ incidence in patients infected with COVID-19 (32).

Females also showed relatively higher frequency of HZ infection than males. Previous studies investigating the sex-based HZ burden have reported mixed findings, with some also identifying a higher incidence of HZ in females and others finding no such difference (28, 33–37). This finding may be attributable to the demographic and clinical profile of the studied population, or health-seeking behavior, rather than a biological contribution (27).

History of known HZ vaccinations was low among the study population (7.1%). This is consistent with previous studies of acceptability of HZ vaccination in Saudi Arabia, in which only 25–53% of study participants were willing to receive the vaccine (21, 38), and less than 10% reported receiving the vaccine (21). The low uptake of HZ vaccination may be explained by the lack of awareness of the impact of HZ and availability of HZ vaccination previously reported amongst the general public in Saudi Arabia (19–22). For example, a recent study of public perception toward HZ and its vaccination in Saudi Arabia reported that over half of respondents were not aware that HZ vaccination is provided by the Saudi Ministry of Health for certain populations (19). Additional previously-identified reasons for poor HZ vaccination uptake in Saudi Arabia include hesitation due to concerns of vaccination costs and possible side effects, a perception of being in good health, and a lack of knowledge of the impact of HZ vaccination (22, 39, 40). This demonstrates that there is unmet need for general HZ and HZ vaccination awareness in Saudi Arabia. Further studies on HZ vaccination knowledge, attitudes, and practices, along with vaccination impact studies in Saudi Arabia, may be one method for achieving this.

The most common HZ-related complication in this study was PHN (17.6%) which was within the range reported by previous studies (5–30%) (3, 41–43). When investigating the characteristics that were predictors of developing complicated HZ, age ≥60 years, autoimmune conditions, medically-diagnosed depression, and chronic lung diseases were identified as significant. Additionally, age ≥50 years, organ transplantation, and coronary artery disease were identified as significant predictors of developing PHN. This is in line with published data that have shown the association of severe or recurrent HZ with several health conditions and associated medications, including cancer, autoimmune diseases, rheumatoid arthritis, and inflammatory bowel disease (44–48). Identifying risk factors is vital to reduce the burden of HZ among target populations through vaccination programs and early intervention with antiviral medications (27).

In the context of healthcare resource utilization, the present study attempts to describe the general HZ patient pathway to better contextualize the resource utilization and cost endpoints of patients with HZ in Saudi Arabia. The burden of HZ on healthcare resources in this study was significant, with 12.5% of cases resulting in hospital admission with a mean length of stay of 10.1 days. A further 9.1% of cases required airborne isolation, which from clinical practice, is often required for patients with disseminated disease or until disseminated disease is ruled out for those who are immunocompromised (49). Although it might be expected that outpatient visits would have a larger associated cost than medications and hospitalizations (as this is the typical starting point of the patient pathway), this study reported medications to have a greater cost, illustrating that certain patients were prescribed medications through different channels (i.e., directly through emergency department visits or through Family Medicine). This raises the concern of potential underestimation of both prevalence of cases and costs, with previous reviews of HZ epidemiology acknowledging that underreporting is a limitation of using retrospective databases (3, 6). There is, therefore, a need for prospective surveillance studies to truly capture the burden of HZ in Saudi Arabia. Additionally, improving reporting mechanisms for HZ across healthcare sectors in Saudi Arabia may help to improve the accuracy of these future epidemiological studies. Although HZ is now included in the list of notifiable diseases in Saudi Arabia, there is still a need to effectively disseminate information regarding HZ among healthcare providers to enhance their ability to accurately identify and report HZ (23). This is also true of other countries, including Qatar and Bahrain, where HZ is a notifiable disease but there remains a lack of HZ surveillance due to various infrastructural and social factors (24, 50, 51).

This study also found that a relatively high proportion of patients with HZ were diagnosed through emergency department visits. This may reflect the increased inappropriate utilization of emergency department by conditions that can be managed in PHCs. Several reasons for patient self-referral to emergency departments in Saudi Arabia have been discussed in a previous cross-sectional study, and include a perception of urgency, easy access, and perceived limited resources at PHCs (52). This provides valuable insights into the HZ patient pathway in Saudi Arabia, demonstrating that patients may not enter this pathway through an outpatient setting due to ease of access, instead entering the pathway via an emergency department visit. These patients may be discharged with a medication prescription, meaning they do not incur the same utilization or costs as inpatients. Some patients may have follow-up appointments at private hospitals, where costs are covered by the patient or by their medical insurance.

This study also estimated the mean total cost per case of HZ without PHN to be slightly higher than that of HZ with PHN. In contrast, previous studies have estimated the mean cost of HZ with PHN cases as higher than that of HZ cases without complications (53, 54), with one study of the population-based burden of HZ finding HZ with PHN contributed to 38.5% of total costs of HZ (55). This may reflect the likely underestimation of PHN-related costs in the MNGHA hospitals and affiliated PHCs included in the present study, as the long-term impact (i.e., recurrences, sequalae, treatment of associated chronic conditions) subsequent to PHN was not captured. In addition, any long-term treatments outside of the MNGHA hospital system, such as out-of-pocket payments, were not captured in this study. However, median total cost per case of HZ without PHN was estimated to the slightly lower than that of HZ with PHN, which is more reflective of previous studies (53, 54).

While the current study provided an estimate of the direct cost associated with management of HZ disease, the study was limited in that the indirect cost related to increased burden on the healthcare system, loss of productivity, debilitating pain, and impact on quality of life was not assessed (56–59). Estimating indirect cost associated with illness and disease has several established caveats; these include methodological differences that cause wide variation in cost estimates between studies and evidence gaps that hinder comprehensive analyses of these costs (4, 60, 61). Additionally, the generally older age of the population included in this study may have meant that their employment status and economic contributions were lower than the general population, further complicating measuring indirect costs in this population. Because of these limitations, indirect cost of HZ disease was not considered in the analyses of this study and, therefore, actual total costs (including indirect costs) may be higher than estimated in this study. Moreover, costs associated with mild cases and long-term consequences were also not assessed, leading to potential underestimation of overall direct medical cost associated with HZ. It is also worth noting that the healthcare service for eligible populations in the MNGHA hospitals is free, and although the estimate provided in this study is based on actual cost, this is much lower when compared to the other health sectors in Saudi Arabia and its surrounding countries.

This study may also be limited because of missing data and because the extent of miscoding, non-coding, and non-reporting of mild cases is not known. However, the latter is anticipated to be minimal as the data were verified using two sources of information (i.e., electronic medical records and reported data to IPC Department). An additional inherent limitation of this study is that the available databases are intended for patient care, and the data are not systematically recorded for research purposes. Furthermore, cost estimates did not include the costs of any laboratory or radiological tests performed during patients’ acute illness or hospital stay, which may have resulted in an underestimation of the total costs. The retrospective nature of the study also meant that the availability of variables is limited to those recorded.

Furthermore, the use of hospital-based data may be more representative of patients with severe HZ disease rather than the broader HZ population, limiting assessment of the burden of HZ across healthcare settings. Additionally, the study population of patients at MNGHA hospitals and affiliated PHCs may also not be representative of the national population of Saudi Arabia, with potential regional variation in socio-demographic and economic factors. Nevertheless, as eligibility for care in MNGHA hospitals and affiliated PHCs may be extended to family members and other non-eligible patients, and eligibility for military personal continues following retirement, the current findings of this study still provide useful insights into HZ epidemiology in Saudi Arabia.

## 5 Conclusion

In this study, we described clinical-epidemiological characteristics of patients with diagnosis of HZ reported to tertiary care hospitals in Saudi Arabia. Patients with older age (≥50 years) or altered immune function were at a higher risk of developing complicated HZ and PHN. These findings may help inform efforts to improve community awareness and preventive interventions targeting those at higher risk.

Future studies should focus on estimating both direct and indirect cost of HZ-related conditions. Moreover, further investigation into factors associated with increased risk of complicated zoster including persistent pain, disseminated zoster, and related hospitalization should also be pursued, as these require enhanced healthcare. Finally, improved public awareness of HZ and HZ vaccination is needed to alleviate the burden of the disease in Saudi Arabia.

## Data Availability

The original contributions presented in the study are included in the article/Supplementary material, further inquiries can be directed to the corresponding author/s.
