# Supplementary material for: Epidemiology of herpes zoster in National Guard Hospitals in Saudi Arabia: a 6-year retrospective chart review study
Source: Front Public Health. 2025 Feb 20;12:1479640. doi: 10.3389/fpubh.2024.1479640 (PMC11882511; doi:10.3389/fpubh.2024.1479640)
Supplement: Supplementary file 1 [file Data_Sheet_1.docx]

SUPPLEMENTARY MATERIALS

# **Supplementary Table 1.** Prevalence of HZ per age group during the study period (2017–2022)

| **Age (years)** | **Number of HZ cases** | **Average population**  **(per age group)^a^** | **Period prevelance  (%)** | **Cumulative incidence (per 1,000 population)** |
| --- | --- | --- | --- | --- |
| <18 | 23 | 283,317 | 0.01 | 0.08 |
| 18–24 | 45 | 82,555 | 0.05 | 0.55 |
| 25–29 | 54 | 66,807 | 0.08 | 0.81 |
| 30–34 | 78 | 70,337 | 0.11 | 1.11 |
| 35–39 | 79 | 57,657 | 0.14 | 1.37 |
| 40–44 | 61 | 46,280 | 0.13 | 1.32 |
| 45–49 | 58 | 37,911 | 0.15 | 1.53 |
| 50–54 | 87 | 38,557 | 0.23 | 2.26 |
| 55–59 | 140 | 38,000 | 0.37 | 3.68 |
| 60–64 | 115 | 38,506 | 0.30 | 2.99 |
| 65–69 | 90 | 25,612 | 0.35 | 3.51 |
| 70–74 | 64 | 15,071 | 0.42 | 4.25 |
| 75–79 | 63 | 11,637 | 0.54 | 5.41 |
| 80–84 | 33 | 8,186 | 0.40 | 4.03 |
| 85–89 | 20 | 4,324 | 0.46 | 4.63 |
| ≥90 | 9 | 2,989 | 0.30 | 3.01 |

[a] Average annual number of medical records in the studied hospitals during the whole study period (2017–2022), stratified by age. **Abbreviations**: HZ: herpes zoster.

# **Supplementary Table 2.** HZ disease management and medications

| **Variables** | **N=1,019** |
| --- | --- |
| **Hospital admission**;^a^ n (%) | 127 (12.5) |
| Intensive care unit admission | 12 (1.2) |
| Airborne isolation | 93 (9.1) |
| **Length of hospital stay**; days |  |
| Mean | 10.1 |
| Median (IQR) | 6.0 (2.0–14.0) |
| **Outpatient visits (diagnosis and follow-up)**; n (%) |  |
| 1 | 352 (34.5) |
| 2 | 210 (20.6) |
| ≥3 | 134 (13.2) |
| **Antibiotic use**; n (%) |  |
| Systemic | 42 (4.1) |
| Topical | 9 (0.9) |
| Not used | 968 (95.0) |
| **Systemic antiviral medications**;^b^ n (%) |  |
| Acyclovir | 524 (51.4) |
| Famciclovir | 362 (35.5) |
| Valacyclovir | 2 (0.2) |
| Valganciclovir | 4 (0.4) |
| **Topical antiviral medications**; n (%) | 251 (24.6) |
| **Gabapentin/Pregabalin**; n (%) | 211 (20.6) |

[a] Calculated from date of diagnosis (number of patients received systemic antiviral=813; 79.8%); [b] 55 patients (5.4%) received two systemic antivirals. **Abbreviations:** HZ: herpes zoster; IQR: interquartile range.

# **Supplementary Table 3.** Multivariable logistic regression analysis of factors associated with complicated HZ

| **Variables** | **HZ with complications,^a^**  **n (%)** | **HZ without complications,^b^**  **n (%)** | **OR (95% CI)^c^** | **P value** |
| --- | --- | --- | --- | --- |
| Sex; |  |  |  |  |
| Female | 160 (30.7) | 362 (69.3) | 0.85 (0.65–1.13) | 0.26 |
| Male | 159 (32.0) | 338 (68.0) |  |  |
| Aged ≥60 years | 215 (34.6) | 406 (65.4) | 1.42 (1.05–1.93) | **0.03** |
| Hematopoietic stem cell transplant | 8 (44.4) | 10 (55.6) | 1.93 (0.72–3.75) | 0.19 |
| Organ transplant | 9 (39.1) | 14 (60.9) | 1.21 (0.51–2.86) | 0.67 |
| Rheumatoid arthritis | 17 (48.6) | 18 (51.4) | 1.88 (0.93–3.78) | 0.08 |
| Autoimmune disease | 21 (47.7) | 23 (52.3) | 2.45 (1.31–4.58) | **<0.01** |
| Immunosuppressive medications | 45 (36.9) | 77 (63.1) | 0.99 (0.60–1.63) | 0.96 |
| Malignancy | 27 (38.0) | 44 (62.0) | 1.20 (0.70–2.04) | 0.52 |
| Chronic kidney disease | 30 (36.6) | 52 (63.4) | 0.90 (0.54–1.51) | 0.68 |
| Diabetes mellitus | 135 (35.2) | 249 (64.8) | 1.06 (0.77–1.46) | 0.73 |
| Hypertension | 148 (37.4) | 248 (62.6) | 1.35 (1.00–1.89) | 0.05 |
| Depression | 12 (50.0) | 12 (50.0) | 2.68 (1.17–6.11) | **0.02** |
| Dyslipidemia | 45 (35.7) | 81 (64.3) | 1.08 (0.72–1.65) | 0.69 |
| Coronary artery disease | 17 (51.5) | 16 (48.5) | 1.83 (0.89–3.76) | 0.098 |
| Cardiovascular disease | 34 (37.8) | 56 (62.2) | 0.88 (0.54–1.45) | 0.63 |
| Asthma | 31 (41.9) | 43 (58.1) | 1.23 (0.73–2.10) | 0.44 |
| Chronic lung disease | 22 (51.2) | 21 (48.8) | 1.95 (1.04–3.68) | **0.04** |
| Recent COVID-19 infection^d^ | 34 (39.1) | 53 (60.9) | 1.20 (0.85–1.71) | 0.31 |

Blue shaded rows represent significant predictors of complicated HZ (p<0.05). [a] n=319; [b] n=700; [c] Estimated using backward logistic regression method; [d] Median time of HZ infection following COVID-19 infection=261 days (IQR=124–468 days). **Abbreviations:** CI: confidence interval; COVID-19: coronavirus disease 2019; HZ: herpes zoster; OR: odds ratio.

# **Supplementary Table 4.** Multivariable logistic regression analysis of factors associated with PHN

| **Variables** | **PHN** | | **OR (95% CI)^c^** | **P value** |
| --- | --- | --- | --- | --- |
|  | **Yes,^a^ n (%)** | **No,^b^ n (%)** |  |  |
| Sex; |  |  |  |  |
| Female | 92 (17.6) | 430 (82.4) | 0.95 (0.68–1.33) | 0.77 |
| Male | 87 (17.5) | 410 (82.5) |  |  |
| Aged ≥50 years | 123 (19.8) | 498 (80.2) | 1.45 (1.02–2.05) | **0.04** |
| Hematopoietic stem cell transplant | 3 (16.7) | 15 (83.3) | 1.03 (0.27–4.03) | 0.96 |
| Organ transplant | 8 (34.8) | 15 (65.2) | 2.50 (1.04–6.02) | **0.04** |
| Rheumatoid arthritis | 6 (17.1) | 29 (82.9) | 0.86 (0.33–2.20) | 0.75 |
| Autoimmune disease | 9 (20.5) | 35 (79.5) | 1.47 (0.68–3.18) | 0.33 |
| Immunosuppressive medications | 23 (18.9) | 99 (81.1) | 1.10 (0.61–1.98) | 0.75 |
| Malignancy | 15 (14.1) | 61 (85.9) | 0.75 (0.37–1.51) | 0.42 |
| Chronic kidney disease | 15 (18.3) | 67 (81.7) | 0.76 (0.40–1.43) | 0.40 |
| Diabetes mellitus | 77 (20.1) | 307 (79.9) | 1.11 (0.76–1.61) | 0.60 |
| Hypertension | 82 (20.7) | 314 (79.3) | 1.24 (0.85–1.80) | 0.27 |
| Depression | 7 (29.2) | 17 (70.8) | 2.12 (0.86–5.22) | 0.10 |
| Dyslipidemia | 25 (19.8) | 101 (80.2) | 1.04 (0.63–1.72) | 0.89 |
| Coronary artery disease | 11 (33.3) | 22 (66.7) | 2.21 (1.04–4.68) | **0.04** |
| Cardiovascular disease | 15 (16.7) | 75 (83.3) | 0.69 (0.37–1.28) | 0.24 |
| Asthma | 9 (12.2) | 65 (87.8) | 0.60 (0.29–1.24) | 0.17 |
| Chronic lung disease | 8 (18.6) | 35 (81.4) | 1.26 (0.53–2.99) | 0.60 |
| Recent COVID-19 infection^d^ | 19 (21.8) | 68 (78.2) | 1.36 (0.78–2.35) | 0.28 |

Blue shaded rows represent significant predictors of PHN (p<0.05). [a] n=179; [b] n=840; [c] Estimated using backward logistic regression method; [d] Median time of HZ infection following COVID-19 infection=261 days (IQR=124–468 days). **Abbreviations:** CI: confidence interval; COVID-19: coronavirus disease 2019; HZ: herpes zoster; OR: odds ratio; PHN: post-herpetic neuralgia.

# **Supplementary Table 5.** Direct medical costs related to HZ, with and without PHN, and with and without complications

|  | **Medication** | **Outpatient visit** | **Emergency department visit** | **Hospitalization** | **Overall** |
| --- | --- | --- | --- | --- | --- |
| **HZ (Overall; N=1,019)** | | | | | |
| **Utilization**, n | 990 | 1,266 | 671 | 127 |  |
| **Total cost**,^a^ SAR | 582,951 | 305,900 | 147,500 | 1,470,900 | **2,507,251** |
| **Mean cost per case**, SAR  **Median (IQR) cost per case**, SAR | 589  311 (168–1,034) | 440  400 (200–600) | 240  200 (200–300) | 11,582  6,000 (2,000–14,000) | **2,461**  927 (507–1,636) |
| **HZ with PHN (n=179)** | | | | | |
| **Utlilzation**, n | 173 | 299 | 120 | 17 |  |
| **Total cost**, SAR | 97,547 | 73,400 | 27,200 | 162,900 | **361,047** |
| **Mean cost per case**, SAR  **Median (IQR) cost per case**, SAR | 564  324 (156–1,034) | 506  400 (200–650) | 247  200 (200–300) | 9,582  7,000 (4,000–14,000) | **2,017**  1,006 (615–1,773) |
| **HZ without PHN (n=840)** | | | | | |
| **Utilization**, n | 817 | 967 | 551 | 110 |  |
| **Total cost**, SAR | 485,404 | 232,500 | 120,300 | 1,308,000 | **2,146,204** |
| **Mean cost** **per case**, SAR  **Median (IQR) cost per case**, SAR | 594  307 (169–1,034) | 422  400 (200–600) | 238  200 (200–300) | 11,891  5,500 (2,000–14,000) | **2,555**  915 (493–1,611) |
| **Complicated HZ (n=319)** | | | | | |
| **Utilization**, n | 309 | 476 | 214 | 61 |  |
| **Total cost**, SAR | 196,012 | 114,900 | 47,500 | 882,100 | **1,240,512** |
| **Mean cost per case**, SAR  **Median (IQR) cost per case**, SAR | 634  376 (179–1,040) | 491  400 (200–600) | 244  200 (200–300) | 14,461  7,000 (4,000–15,000) | **3,889**  1,239 (611–1,983) |
| **Non-complicated HZ (n=700)** | | | | | |
| **Utilization**, n | 681 | 790 | 457 | 66 |  |
| **Total cost**, SAR | 386,939 | 191,000 | 100,000 | 588,800 | **1,266,739** |
| **Mean cost per case**, SAR  **Median (IQR) cost per case**, SAR | 568  293 (167–1,031) | 413  350 (200–600) | 238  200 (200–300) | 8,921  4,500 (1,700–13,000) | **1,810**  877 (481–1,450) |

[a] Total HZ treatment cost includes PHN treatment cost. **Abbreviations:** HZ: herpes zoster; IQR: interquartile range; PHN: post-herpetic neuralgia; SAR: Saudi Riyal.
